# Normal Distribution of CD8+ T-Cell-Derived ELISPOT Counts within Replicates Justifies the Reliance on Parametric Statistics for Identifying Positive Responses

**DOI:** 10.3390/cells4010096

**Published:** 2015-03-02

**Authors:** Alexey Y. Karulin, Richard Caspell, Marcus Dittrich, Paul V. Lehmann

**Affiliations:** 1 Cellular Technology Ltd., 20521 Chagrin Blvd. Shaker Heights, OH 44122, USA; E-Mails: richard.caspell@immunospot.com (R.C.); pvl@immunospot.com (P.V.L.); 2 Biocenter, University Wuerzburg, Am Hubland, Würzburg 97074, Germany; E-Mail: marcus.dittrich@biozentrum.uni-wuerzburg.de

**Keywords:** ELISPOT, statistics, *t*-Test, ANOVA, normal distribution, T-cells

## Abstract

Accurate assessment of positive ELISPOT responses for low frequencies of antigen-specific T-cells is controversial. In particular, it is still unknown whether ELISPOT counts within replicate wells follow a theoretical distribution function, and thus whether high power parametric statistics can be used to discriminate between positive and negative wells. We studied experimental distributions of spot counts for up to 120 replicate wells of IFN-γ production by CD8+ T-cell responding to EBV LMP2A (426 – 434) peptide in human PBMC. The cells were tested in serial dilutions covering a wide range of average spot counts per condition, from just a few to hundreds of spots per well. Statistical analysis of the data using diagnostic Q-Q plots and the Shapiro-Wilk normality test showed that in the entire dynamic range of ELISPOT spot counts within replicate wells followed a normal distribution. This result implies that the Student *t*-Test and ANOVA are suited to identify positive responses. We also show experimentally that borderline responses can be reliably detected by involving more replicate wells, plating higher numbers of PBMC, addition of IL-7, or a combination of these. Furthermore, we have experimentally verified that the number of replicates needed for detection of weak responses can be calculated using parametric statistics.

## 1. Introduction

T-cell ELISPOT assays measure the antigen-induced reactivation of effector memory T-lymphocytes (T_EM_) via the detection of the cytokines that individual T-cells secrete. In this assay, each antigen-specific T_EM_ cell leaves behind a cytokine footprint, in the form of a spot, which is visualized either by enzymatic or fluorescent means. Counting the positive responses within the total number of PBMC plated therefore establishes the frequency of the antigen-specific T_EM_ cells in the cell sample tested. In addition to testing the antigen-induced spots, the assay requires the inclusion of “medium control wells” as negative controls to establish spot counts when the cells are plated without antigen. Such spots can result from non-T-cells constitutively secreting the cytokine that needs to be distinguished from cognate antigen-induced cytokine production by T-cells. When assay conditions are carefully selected, including the use of serum-free medium, the medium spot counts for IFN-γ, IL-2, IL-4, IL-5 and IL-17 are very low or undetectable for healthy donors [1,2]. Cytokine storms in diseased individuals, however, can result in an elevated medium background, primarily resulting from cytokine production by cells of the innate immune system, such as macrophages, NK cells and dendritic cells. Most of the time, such background spots are smaller than the T_EM_ cell-derived, antigen-induced spots and proper gating strategies are capable of discerning between the two [3]. ELISPOT counts determined without involving the analysis of spot size distributions and proper gating could be unreliable, and subsequently, their statistical analysis may not be suited for detecting T-cell responses.

To establish the presence of T-cell reactivity in an individual, therefore, ELISPOT results are evaluated in terms of spot counts in antigen-containing *vs.* medium control wells. To test for increased frequencies of antigen-reactive T-cells in subject groups, such antigen-specific reactivity levels are compared. For example, when vaccines are tested, the frequency of antigen-specific T-cells in PBMC is tested prior to vaccination, as well as after, and vaccinated subject groups are compared with control (unimmunized or placebo) groups. The basis of all of these comparisons is, however, the precise determination of the frequency of the antigen-specific T-cells in each subject, as determined by the difference between (properly gated) spot counts in replicate wells of the medium control *vs.* antigen-induced wells.

Because T-cells in the blood encompass millions of antigen-specificities, those specific for a given antigen often occur in low frequencies, even after they have undergone clonal expansion. ELISPOT tests therefore need to reliably detect T-cells that occur in low frequency in the blood, resulting in moderately elevated spot counts in antigen-stimulated wells *vs.* those in medium control wells. After being initially employed as a basic science research tool, during the past decade, T-cell ELISPOT has become widely used for immune monitoring in various clinical fields, and several of these fields independently adopted different acceptance criteria for defining the cut-off between positive and negative responses. Criteria were selected considering the possible false results of the test. Some of these tests require high specificity (e.g., vaccine testing), ensuring low rates of false positive results, whereas others need high sensitivity (*i.e.*, disease diagnostics where false negative results are a greater danger). Biologically-relevant protective responses could be weak in ELISPOT, yet clinically highly significant, requiring positive response detection criteria with high sensitivity. This is particularly relevant when measuring anti-tumor CD8+ T-cell responses with ELISPOT.

All acceptance criteria for T-cell responses can be divided into two major categories: empirical and statistical. Empirical criteria most often are based on a combination of the minimal number of spots detected and a “fold difference” between antigen-containing and control wells. For example, an empirical criteria widely used in the HIV field states that an IFN-γ ELISPOT response to an HIV peptide pool is positive if the average number of spots per 2 × 10^5^ PBMCs is higher than 10 and at least four-fold the average number of spots in negative wells [4]. Empirical criteria, however, are justified only when they are correlated with the clinical outcomes [5].

In most areas of immune monitoring, however, there is still no known correlation between the disease-protective effect and the magnitude and the Th1/Th2/Th17/polyfunctional quality of the T-cell response [6]. Rather, the goal of immune monitoring is to identify whether a T-cell response has been engaged in the test subject. These data on T-cell immunity can then be correlated with clinical outcomes. Such a correlation, in turn, must be established using appropriate statistical methods. When such a correlation cannot be established, objective statistical criteria must be applied. In this case, it is not possible to make conclusions about the biological relevance of particular treatments based on the ELISPOT results. Rather, with a defined probability (usually 95%), it can be established whether spot counts in antigen-containing wells and control wells are statistically different, that is whether a T-cell response has been engaged.

Since the presence or absence of a T-cell response is reflected in the frequency of antigen-specific T-cells, statistical methods should be applicable to unambiguously identify an antigen-specific response. In statistical analysis, the significance of the result is expressed in terms of “null” hypothesis testing. The “null” hypothesis states that any difference that occurs between the means of two groups (in this case, spot counts in replicate medium control wells *vs.* spot counts in antigen-stimulated replicate wells, after proper gating) is due to random errors. The level of significance (α) defines the probability of a Type 1 error rejecting the “null” hypothesis and, thus, reporting a significant difference when there is none. The confidence level (1 − α) × 100% is the probability of accepting the “null” hypothesis when it is true. A Type 2 error is the probability (β) of accepting the “null” hypothesis when it is not true and, thus, missing the existing difference. The probability of rejecting the “null” hypothesis when it is not true is called the “power” of the test and is equal to (1 − β) × 100%. Thus, the test significance (α) is similar to the specificity and defines the rate of false positive results. The power of the test (1 − β) is similar to the sensitivity and defines the rate of false negative results. Significance and power are in the same reciprocal relationship as the sensitivity and specificity of any bioassay. When a statistical test is performed, the level of significance α (usually 0.05) is set by the investigator.

All statistical methods used in the ELISPOT field can, in turn, be divided into two major categories: parametric and non-parametric. Parametric methods (e.g., *t*-test, ANOVA) generally have higher statistical power, but are appropriate only if the ELISPOT counts within replicate wells follow a known theoretical distribution function (for example, a normal distribution). Among non-parametric methods used for ELISPOT, we should mention the Wilcoxon rank-sum test (the non-parametric analog of the *t*-test) [7] and different variants of bootstrap (permutation based) strategies [8] including distribution-free resampling (DFR) [9,10,11,12]. Non-parametric methods do not require that the distribution is known, and they typically show a high level of specificity (the rate of false positives is low), but a low power (the rate of false negative results is high) [13].

It has been argued that parametric methods may have higher rates of false positive results in multiple comparison tests (known as family-wise error) when, for example, several antigens are tested for the same donor [11]. However, there are several efficient ways to correct for the rate of false positive responses in multiple comparisons [14,15,16,17], and there are examples when such corrections were used for ELISPOT data [18,19]. We have limited the scope of this study to single comparison tests, where, for each given donor, the response to each antigen is compared to the corresponding medium control.

To establish whether ELISPOT counts in replicate wells follow a certain theoretical distribution, one should test a high number of replicate wells, ideally more than 100 wells. In addition, a wide range of cell numbers should be tested, covering the entire spectrum of spot counts: from a few to hundreds of spots per well. The amount of cell material needed for this type of a study makes it prohibitive when testing patient samples. Patient material is typically tested in triplicate wells for both antigen and medium. In the absence of data showing whether or not ELISPOT counts in replicate wells are normally distributed, non-parametric tests have been favored [11], but as stated above, these have a high rate of false negative results. When the sensitive detection of T-cell reactivity is an issue, the ability to use high power parametric statistics becomes crucial.

This study addressed the issue of ELISPOT data distribution and experimentally tested the implications. In a preceding approach, we used IFN-γ constitutively expressing transfected CHO cells and found that ELISPOT counts in replicate wells followed a normal distribution over a wide range (except for counts below 20 spots per well, where the Poisson distribution showed a somewhat better fit) [13]. This simplified approach allowed us to model ELISPOT measurements when cytokine production is constitutive and independent of antigen-presenting cells (APC). Requiring the antigen-specific T-cells to become activated on APC, however, might produce more complex results. We therefore extend our study here to antigen-specific T-cell ELISPOT, testing the HLA-A2-restricted CD8+ T-cell IFN- response to a dominant peptide of human Epstein-Barr virus, EBV LMP2A (426 – 434), using a large number (120) of replicate wells for several serial dilutions of PBMC. The data clearly show that starting from an average of six spots per well and up to 350 spots per well, antigen-specific CD8+ T-cell-derived ELISPOT results also follows a normal distribution function. The presence of normally-distributed data, in turn, means that parametric tests, such as the *t*-test or ANOVA, can be used to establish with high confidence the difference between replicates of medium control wells and antigen-containing wells.

If spot counts are normally distributed and the well-to-well variance is known, the significance level, power of the test and number of replicates are in a known relationship. Therefore, predictions can be made on how many replicates should be used and, most important for practical use, how to minimize the number of replicate wells while still obtaining statistically-significant results. These predictions were tested experimentally using weak ELISPOT responses from two HLA-A2 EBV-positive donors to a subdominant HLA-A2-restriced EBV BMLF1 (259–267) peptide. Both donors were seropositive to EBV and had a strong IFN-γ response to LMP2A (426–434). We found that theoretical predictions based on a normal distribution closely matched the experimentally-defined numbers of replicate wells necessary to discriminate these responses from medium controls. We also showed experimentally that plating higher numbers of PBMC per well and adding IL-7 to the wells can be used to detect weak reactivity with fewer replicates and that increasing the number of replicates using fewer PBMC per well accomplishes the same goal with much higher efficiency in cell utilization.

## 2. Experimental Section

### 2.1. Cells

Cryopreserved human PBMC of 2 HLA-A2-positive donors were acquired from a library of PBMC (ePBMC, CP1, CTL, Shaker Heights, OH, USA). In addition to high-resolution HLA-typing, these PBMC had been previously characterized for T-cell reactivity to a panel of antigens. Prior to testing, the PBMC cryovials, stored in the liquid N_2_ vapor phase, were transferred to dry ice in Styrofoam containers for transport to and short-term storage in the laboratory. The cells were thawed following a protocol established to provide the optimal recovery and functionality for cryopreserved PBMC [2]. Specifically, to rapidly warm up to 37 °C, the cryovials were placed for 8 min in a 37 °C glass bead bath (CTL-BB-001). The cryovials were inverted twice to re-suspend the cells, and the 1-mL cell suspension contained in each cryovial (10 million cells) was gently aspirated utilizing a wide-bore 2-mL pipette and transferred into a 15-mL V-bottom Falcon tube. To recover the residual cells, the cryovials were rinsed by adding 1 mL 37 °C warm CTL Anti-Aggregate Wash™ medium (CTL-AA-005) containing benzonase. An additional 8 mL CTL Anti-Aggregate Wash™ medium at 37 °C was added to the 15-mL tube at a rate of 2 mL per 5 s. Before washing, an aliquot of these PBMC was counted by fluorescence microscopy using Acridine orange and ethidium bromide (AO/EB) to stain live and dead cells, respectively. PBMC were washed twice in 10 mL CTL-Test™ medium (CTLT-005) and re-suspended at a final concentration of 3 × 10^6^ PBMC/mL in the same medium. The freshly-thawed PBMC were plated onto an ELISPOT assay plate (Msn HTS IP, Millipore, MA, USA) within 1 h of thawing. The IFN-γ-expressing CHO cells were generated in our laboratory [13].

### 2.2. ELISPOT

Human Interferon-γ ImmunoSpot® kits (CTL-H1FNG-1/5M) were obtained from CTL. The assay was performed according to the manufacturer’s recommendations. The antigens (EBV-dominant peptide LMP2A (426 – 434) and subdominant EBV BMLF1 (259 – 267) (EZ Biolab Inc., Carmel, IN, USA)) were plated at 4 μg/mL first into the capture antibody-coated assay plate in a final volume of 100 µL per well. The antigens were dissolved in CTL-Test™ medium (CTLT-005). In a previous work, we observed that adding IL-7 can improve the signal-to-noise performance of ELISPOT assays [20]. Therefore, where specified, the IL-7-supplemented CTL-Test Plus™ medium was used (CTLTP-005). These media, without dissolved antigen, constituted the medium negative controls. The plates containing the antigens and medium controls were stored at 37 °C in a CO_2_ incubator until the cells were ready for plating. Thawed PBMC were adjusted in CTL test medium to the required concentrations, and 100 µL/well were plated using wide-bore pipette tips. Plates were gently tapped on each side to ensure even distribution of the cells as they settled and incubated for 24 h at 37 °C in a CO_2_ incubator. Following completion of the ELISPOT protocol, the plates were air dried in a laminar flow hood prior to analysis.

ELISPOT plates were scanned and analyzed using an ImmunoSpot® S6 UV Reader by CTL. Spots were counted automatically by using the ImmunoSpot® v.5.3 Software (Basic Count™ mode) for each antigen stimulation condition and the medium negative controls.

### 2.3. Statistical Evaluation

To analyze the empirical distributions of ELISPOT data, the Shapiro-Wilk normality test was used in combination with analytical Q-Q plots. To detect positive responses over medium controls, the Student’s two-sided *t*-test was used. In all cases (unless specified otherwise), statistical tests were performed with a significance level α = 0.05. Statistical analysis was carried out using the SPSS [21] and XLSTAT [22] software suites.

## 3. Results and Discussion

### 3.1. ELISPOT Counts Are Linear in the Range between 2.5 × 10^4^ and 10^6^ PBMC Plated per Well

**Figure 1:**
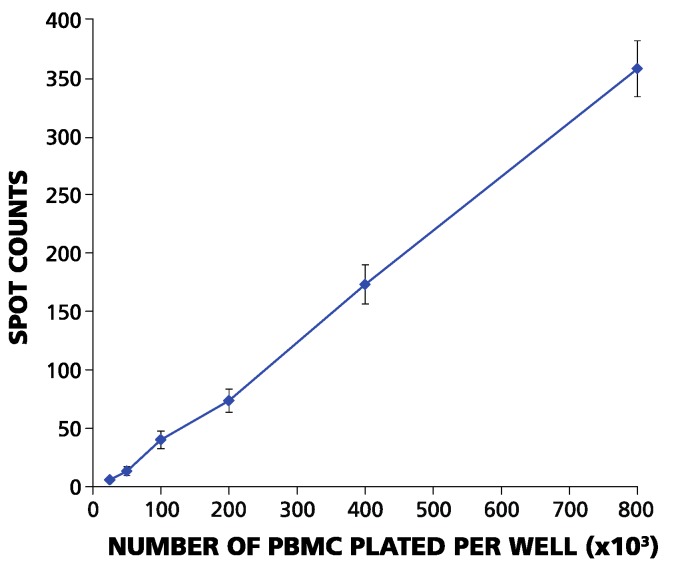
ELISPOT counts and plated PBMC numbers have a linear relationship in the range between 2.5 × 10^4^ and 10^6^ PBMC per well. The PBMC of an HLA-A2-positive donor were plated for each of the cell numbers specified on the X-axis per well, with 120 replicates for each cell number. The mean number of IFN-γ spots elicited by EBV peptide LMP2A (426–434) from these 120 replicate wells is shown on the Y-axis. Error bars represent ± SD.

To become activated by an antigen, T-cells must make contact with APC. Therefore, the numbers at which PBMC are plated will affect the results: when plated at less than 5 × 10^4^ PBMC per well in a 96-well plate, the cells no longer form a monolayer, and recall responses become undetectable [23]. On the other hand, when plated at more than 10^6^ PBMC per well, the cells become crowded and settle in several layers, also leading to a loss of accurate ELISPOT counts. We systematically studied spot counts within these two extremes. We performed serial dilutions of PBMC that exhibited strong CD8+ T-cell reactivity to LMP2A (426–434), with cell numbers ranging from 2.5 × 10^4^ to 10^6^ cells per well, using 120 replicative wells. The resulting spot counts (between six and ~350 per well) displayed a linear relationship to the numbers of PBMC plated over this range (Figure 1). The linear relationship between the numbers of PBMC plated and spot numbers eliminates the possibility of systematic errors resulting from cell counting, dilution or plating. Therefore, any errors in ELISPOT counts can be considered random.

### 3.2. ELISPOT Counts between Six and 350 per Well Follow Normal Distribution Function

**Figure 2:**
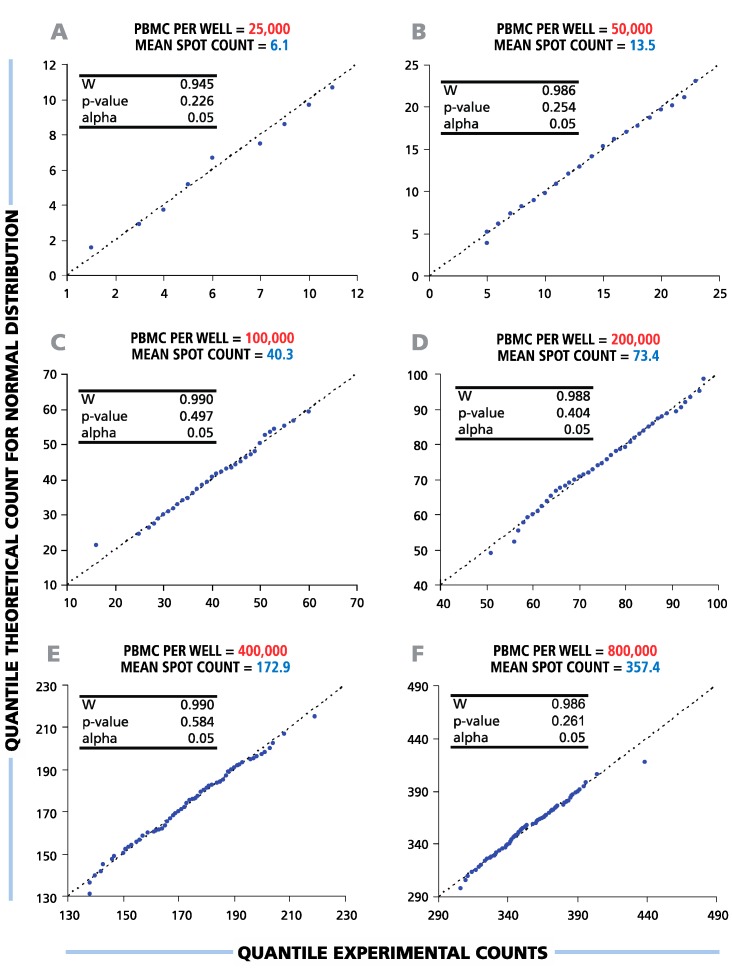
Spot counts between six and 350 per well follow a normal distribution function. Therefore, parametric statistical tests (*t-*test, ANOVA) can be used to discriminate between negative and positive responses over the full range of potential ELISPOT assay results. A representative experiment with a single HLA-A2-positive donor’s CD8+ T-cell IFN-γ response to EBV peptide LMP2A (426 – 434) is shown. For each cell number plated per well (**A**–**F**), the distributional properties of the spot counts were analyzed in 120 replicate wells using diagnostic plots (Q-Q plots) and Shapiro-Wilk statistical normality tests with a significance level of α = 0.05 (shown in the picture inserts). The X-axis labels the quantile experimental counts; the Y-axis labels the quantile theoretical counts for the normal distribution. “W” is the calculated Shapiro-Wilk statistics.

To study the distribution function of ELISPOT counts in replicate wells, the results of the serial dilutions shown in Figure 1 were subjected to statistical evaluation. To test the hypothesis of a normal data distribution, we used diagnostic (Q-Q) plots and Shapiro-Wilk normality tests with a significance level α = 0.05 (Figure 2). The Shapiro-Wilk null hypothesis states that samples come from a normally-distributed population. This test is optimal for sample sizes between 20 and 2000 and was shown to have higher power than other standard normality tests [24,25]. The Q-Q-plots generated for all cell dilutions tested were close to linear, suggesting that spot counts in repetitive wells follow a normal distribution function. *p*-values calculated by the Shapiro-Wilk test were above the set level of significance for all cell dilutions (see the inserts in Figure 2), allowing us to accept the null hypothesis about the normal spot count distribution. Therefore, in the entire practical range of cell numbers that can be tested in T-cell ELISPOT assays, the data in the repetitive wells were found to be normally distributed.

### 3.3. Theoretical Predictions and Experimental Data for the Number of Necessary Replicates Closely Match

**Figure 3:**
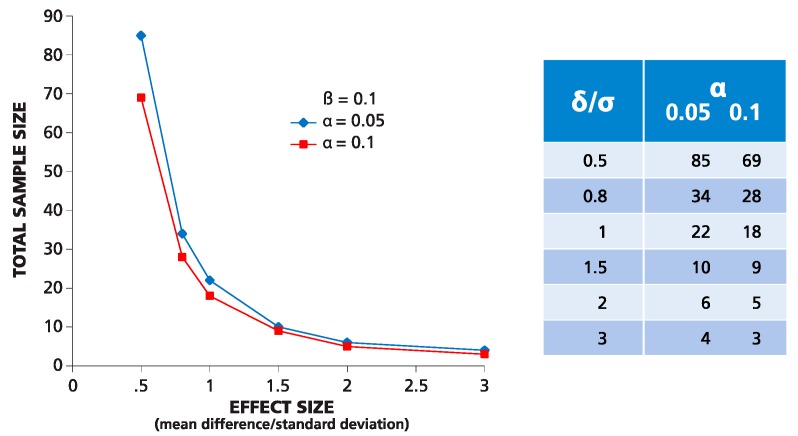
Theoretical prediction for the number of replicate wells required for detecting the difference between two means expressed by “folds” of σ. The numbers of replicates (Y) are calculated in a two-tailed test for a given effect value = δ/σ (X) for two levels of significance α equal to 0.05 and 0.1 and power β set to 0.1.

As ELISPOT counts are normally distributed, parametric statistical methods, such as the Student’s *t*-test, can be used to compare mean values of positive (peptide-containing) and negative (medium control) wells. For normally-distributed data, the variance, level of significance, power and sample size are in a known relation. Therefore, the sample size (number or replicates) required to detect an existing difference between positive and negative wells can be predicted when the standard deviation (σ), level of significance (α) and power (β) are known. In statistics, the difference between two means, μ_1_ and μ_2_, is often expressed as folds of standard deviation δ/σ (effect size), where δ = μ_1_ − μ_2_. Sample size calculators for normal distribution are freely available online, e.g., [26]. Figure 3 shows an example for such calculations using the Student’s *t*-test with the power set to 90%, and two levels of significance: 5% and 10%. As follows from the graph, to detect a mean difference equal to 2σ, with α = 0.05 and β = 0.1, six repetitive wells are needed. It is important to mention that such predictions are possible only in parametric statistics when the distribution function is known.

We set out to test experimentally whether these predictions are true for ELISPOT assays by comparing theoretical curves (similar to those shown in Figure 3) with experimental data generated using IFN-γ-transfected CHO cells. As every transfected CHO cell secrets IFN-γ, one can generate any number of spots per well, including the simulation of various background levels. As an example of such a simulation for generating specific spot counts over different background levels, a certain number of transfected CHO cell was plated in “medium control wells” to create known artificial background counts. For the corresponding “positive wells”, additional CHO cells were added, creating defined positive responses over that known background.

**Figure 4:**
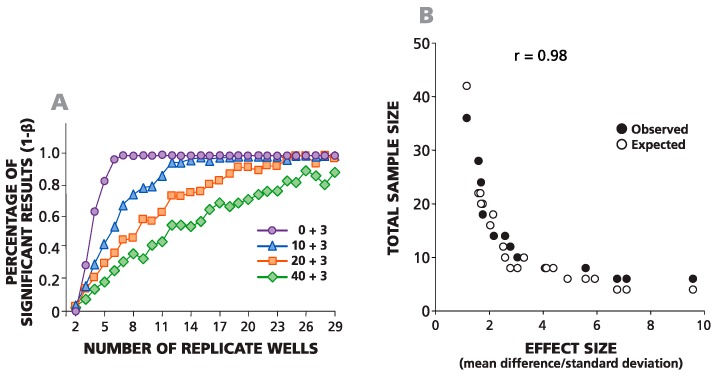
Observed and expected numbers of replicate wells required for detecting a statistically-significant difference between spot counts in positive and negative wells match closely. (**A**) The percentage of statistically-significant positive responses is plotted *vs.* the number of replicative wells for four different, artificially-created weak positive responses (zero background spots plus three spots, 10 plus three, 20 plus three and 40 plus three). The percentage of significant results was calculated for different numbers of replicate wells (from two to 29) generated using permutations of all 29 experimental replicate wells. (**B**) The theoretically-predicted (expected) and experimentally-observed numbers of replicates, necessary for a percentage of significant results over 90% in a two-tailed test, are plotted *vs.* the effect value (δ/σ).

In Figure 4A, an example of such a simulation is shown where negative wells contained on average 0, 10, 20 or 40 spots, and the average difference between the spots in negative and positive wells was always equal to three spots. For one data point, the background was set to be 10 spots (by plating an average of 10 cytokine-producing CHO cells per well), and the experimental wells were set to be 13 spots (by adding an average of 13 cells per well). The same numbers of spots in negative wells (0, 10, 20 and 40) were used to simulate positive responses with spot counts equal to 6, 12, 24 and 48 over the background. The total number of replicates for each condition was 29. To define the minimal number of replicates required for the detection of these positive responses with α = 0.05 and β = 0.1, we used all possible combinations (permutations) of the numbers of wells *n* = 1, 2, 3, 4, *etc.*, up to 29, while testing each permutation in the Student’s *t*-test. The percentage of significant positive results for each sample size is shown in Figure 4A. The number of replicates needed for at least 90% significant results (power 1 − β = 0.9) was used to create an experimental sample size prediction graph. This experimental (observed) and the theoretical (expected) data sample prediction graphs are superimposed in Figure 4B. A strong correlation was observed between experimental numbers and theoretical predictions (coefficient of correlation *r* = 0.98). As follows from the graph, to detect three spots over a background of 10 spots, one needs about 11–13 replicates, and to detect three spots over a background of 20, roughly 20–23 replicates are needed.

### 3.4. Using More Cells per Well Reduces the Number of Replicate Wells Required for the Detection of Weak Responses

**Figure 5:**
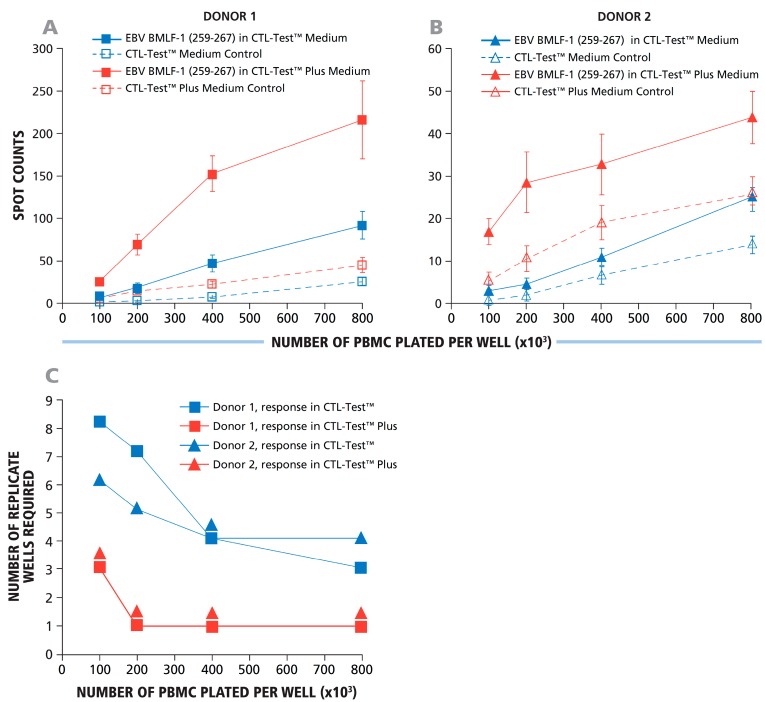
Using more cells per well reduced the number of replicate wells required to detect weak subdominant responses. Using IL-7 containing signal-enhancing CTL-Test Plus™ medium further increased the signal-to-background ratio to the point where even a single well suffices to reveal a positive response, even at low cell numbers. A representative experiment using two HLA-A2-positive donors with a dominant CD8+ T-cell IFN-γ response to EBV peptide LMP2A (426–434) and a weak positive response to the subdominant EBV BMLF1 (259–267) is shown. PBMC were plated at 1, 2, 4 and 8 × 10^5^ cells per well, as specified on the X-axis. (**A**, **B**) The responses for Donors 1 and 2 against subdominant EBV BMLF1 (259–267) are shown as the numbers of spots counted *vs.* the numbers of PBMC plated per well. The assay was performed using standard CTL-Test™ medium and with signal-enhancing CTL-Test Plus™ medium with or without antigen, as shown in (A) and (B), respectively. The mean spot counts and SD for 12 replicate wells for each condition are shown on the Y-axis. Different numbers (from two to 12) of replicates were analyzed using a permutation analysis (similar to the one used in Figure 4) in a two-sample Student’s *t*-test. Minimal required numbers of replicate wells to detect a response with α = 0.05 at different PBMC numbers are shown in (**C**).

To extend the results obtained with transfected CHO cells to the detection of actual antigen-specific T-cell responses, we selected two confirmed EBV HLA-A2-positive donors who showed a strong response against the dominant EBV peptide LMP2A (426 – 434) and studied their CD8+ T-cell IFN-γ response to the subdominant EBV peptide BMLF1 (259 – 267), which is also HLA-A2 restricted. With a high probability, both donors theoretically should respond to this peptide. The goal of this experiment was to determine under which conditions these subdominant responses could be detected with statistical significance. As the factors affecting assay variance within replicate wells result from pipetting errors and spot counting accuracy, these random errors within replicates should be independent of the specific donor-antigen combination. The PBMC were tested at 1, 2, 4 and 8 × 10^5^ cells per well, with 12 replicates for each cell number. Panels A and B in Figure 5 show the responses of Donors 1 and 2, respectively, with and without added human recombinant IL-7 as an assay enhancer in the CTL Test Plus™ medium [20]. When 12 replicates were used, both donors had a detectable IFN-γ response to BMLF1 (259 – 267), over the medium control background. To determine the minimal number of replicates needed to reveal this response with statistical significance, data permutation analysis was carried out as described above for Figure 4B. Panel C in Figure 5 shows a graphical representation for the minimal number of replicates required to detect these weak responses by the Student’s *t*-test with the level of significance α = 0.05. With 10^5^ cells plated, eight and six replicate wells were required for Donors 1 and 2, respectively. The data show that for Donor 1, the positive response became statistically significant when eight replicates were used for both antigen- and medium-containing wells. For Donor 1, therefore, 1.6 × 10^6^ PBMC were required to reveal the positive response (two conditions multiplied by eight replicates multiplied by 1 × 10^5^ PBMC/well). At 4 × 10^5^ cells per well, 4–5 replicates were needed (with five replicates for both conditions requiring 4 × 10^6^ PBMC). The number of replicates needed was reduced to 3–4 at 8 × 10^5^ cells per well (with four replicates for both conditions, requiring 6.4 × 10^6^ PBMC). These data also show that increasing the number of PBMC plated decreases the number of replicates needed to detect weak responses. However, the goal of using fewer replicates is accomplished with far higher economy of PBMC usage when the number of replicates is higher and fewer cells are plated.

### 3.5. The Relative Experimental Error Decreases with Increasing the Number of Spot Counts per Well

**Figure 6:**
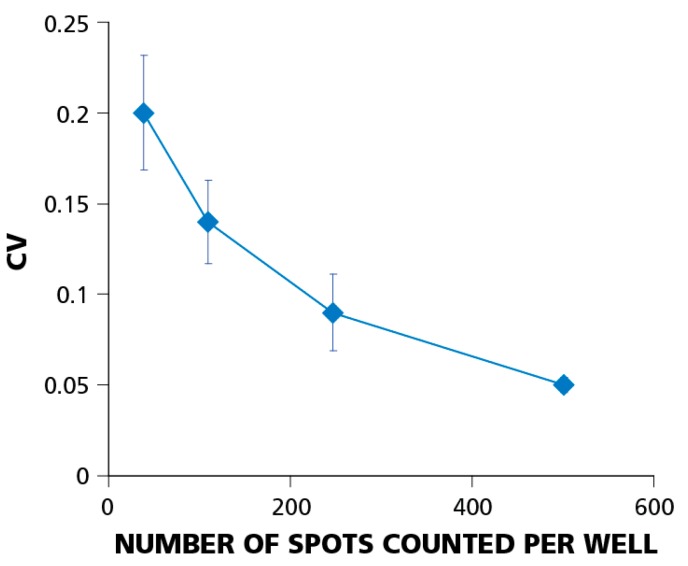
The relative experimental error (coefficient of variation, CV) decreases with increasing number of spot counts per well. Testing PBMC at higher cell numbers helps establish positivity when statistical analysis is at the limit of confidence. CV data were obtained from eight independent experiments using a single donor with a dominant CD8+ T-cell IFN-γ response to EBV peptide LMP2A (426–434). Error bars show ±σ between individual experiments. In individual experiments, CVs were calculated for each of four cell dilutions (5 × 10^4^; 1 × 10^5^; 2 × 10^5^; 4 × 10^5^) using 12 replicate wells for each dilution.

The coefficient of variation (CV) or relative experimental error is calculated as the standard deviation divided by the mean value (σ/μ). Using the relative, rather than the absolute, error allows for comparisons of the well-to-well variation at different cell dilutions. Figure 6 shows how the CV depends on the number of PBMC plated. The figure summarizes data from eight independent serial dilution experiments, each done with 12 replicate wells for each cell dilution. Error bars on the graph represent the standard deviation of the CV. The data imply that the relative well-to-well error decreases with increasing the number of cells plated. At 5 × 10^4^ cells per well, the CV was around 20%; at 4 × 10^5^ cells per well, it dropped to about 5%. This finding explains why using higher cell numbers requires fewer replicate wells for detecting weak responses.

### 3.6. Discussion

In ELISPOT assays, each spot is the signature of an individual analyte-secreting cell. In T-cell ELISPOT assays, therefore, the frequency of antigen-specific T-cells in the blood is measured at the single-cell level. The spot size distribution in ELISPOT wells allows the identification of T-cell-derived cytokine *vs.* background noise resulting from the secretory activity of cells of the innate immune system (or from non-specific substrate precipitation and other artifacts that can contribute to noise). Establishing firm and objective spot counts for each well, therefore, must be the first step of ELISPOT data analysis, before any subsequent statistical analysis of these numbers becomes meaningful [3]. Our present study on the spot count distribution within replicate wells starts at this point, after correct spot counts per well have been firmly established.

One of the primary reasons why ELISPOT has succeeded in many different fields of immune monitoring (vaccine trials, tumor and transplant immunology, autoimmunity) is that the assay allows the investigator to detect rare antigen-specific T-cells among all of the other lymphocytes with irrelevant antigen specificity. Furthermore, the data are at the single-cell level. When the frequencies of antigen-specific T-cells are in the medium range, e.g., at 1/10^4^, one can expect to detect 100 spots within 10^5^ PBMC plated, with a medium background of fewer than 10 (properly gated) and, many times, zero spots. Frequently, such clear-cut results are obtained with a low background and high signal-to-noise resolution [27]. There is no need for statistical proof when positive wells show 10–200-fold spot numbers over negative controls. For strong positive responses, duplicate wells should suffice (for example, to rule out a systematic error when cells or antigens are plated). The Chebyshev theorem defines an interval outside which practically no statistical evaluation is required to prove the difference between positive and negative wells. It states that for any k = 1, 2, 3,… *n*, the probability (P) for the mean value (μ) to be in the range of ±k standard deviations is: (μ – k × σ ≤ μ ≤ μ + k × σ) is equal or higher than (1 − 1/k^2^), where σ is the standard deviation of the mean. Therefore, for the commonly-accepted level of significance σ = 0.05 the probability P = 0.95 = (1 − 1/k^2^), and the minimal effect k = ±4.72 × σ. In other words, with 95% (double sided) probability, positive responses are guaranteed to be different from the negative control if their mean values are at least 4.72 × σ apart. This simple universal criterion is similar to the “three standard deviations” rule, the difference being that the Chebyshev inequality does not require knowledge of the data distribution function (whereas the 3σ rule assumes a normal distribution). What is common for both is the low power to detect weak true positive responses. When the rate of false negative results is not an issue, but false positive results are highly undesirable (testing of vaccines is efficient only when a strong response is protective), the Chebyshev inequality could be very useful and universal.

Parametric statistics becomes essential when it is important to distinguish with high power (sensitivity) between weakly positive and negative results. In this study, we present (to our knowledge, for the first time) statistically-validated evidence that ELISPOT counts obtained from human PBMC for antigen-specific IFN-γ responses of CD8+ T-cells follow a normal (Gaussian) distribution. This notion justifies the use of parametric statistics for the discrimination between negative and positive well counts.

The normal distribution of ELISPOT counts allows answering two other important questions: how many replicates should be used in a particular ELISPOT experiment, and how does the number of replicates depend on the number of cells plated? First, using transfected CHO cells, we experimentally created various levels of “background” spots and increased that number slightly by adding known numbers of CHO cells to create “weakly positive experimental wells”. The predicted and measured numbers of replicates needed to detect these modeled weak responses by using the Student’s *t*-test matched, verifying the suitability of the predictions. As an example of such analysis on PBMC, we used weak CD8+ T-cell responses of healthy HLA-A2 donors against the subdominant EBV determinant, BMLF1 (259–267). Furthermore, in this case, the number of replicate wells required for a statistically-significant difference between antigen-induced responses and medium control wells closely matched the theoretical predictions based on the normal data distribution. Translating these findings into practical recommendations, our data suggest that in most cases, using 4–5 replicates should be suitable for detecting even weak positive responses with effects between 2σ and 3σ (depending on experimental assay variance). Increased numbers of replicates and/or of cells plated per well can be used if even higher resolution is needed to unambiguously detect weak responses.

Our data showed that the well-to-well coefficient of variation decreased with increasing numbers of PBMC per well. In other words, for weak responses, the relative experimental error in ELISPOT counts is smaller at higher numbers of cells per well. As a result, the number of required replicates was lower with higher numbers of PBMC plated per well. The relationship between increasing replicates or cell numbers detecting weak responses leads to interesting implications of our analysis. If cells are limiting, spot counts obtained after seeding a lower amount of cells in a higher number of replicates will reach the significance level substantially faster than when only cell numbers are increased. If material and labor costs rather than cells are limiting, increasing cell numbers can be recommended. Both effects are additive and are recommended when neither is limiting, as also is the addition of IL-7 to the culture.

In parametric statistical tests, such as the Student’s *t*-test, the greatest statistical error usually comes from experimental estimations of standard deviations (and, to a much lesser extent, from estimations of mean values), in particular when few replicates are used. Therefore, when a trial is planned using small numbers of replicates (3–4 is quite common), it could be recommended to have standard deviations of negative and positive wells estimated first in a small pilot study that involves more (10–20) replicates. Experimental errors in ELISPOT are primarily caused by cell pipetting and spot counting errors and do not depend much on the particular donor or antigen. Limited pilot studies allow different laboratories to determine the extent of systematic error in that laboratory, so that this can be taken into account, beyond the random, Gaussian variation. Planning for too few replicate wells going into a study can jeopardize the entire study or clinical trial.

This study was restricted to CD8+ T-cells. CD8+ T-cells recognize the antigen on MHC class I molecules that are expressed on all cell types of the body and, unlike CD4+ T-cells, have permissive costimulatory requirements. Therefore, while essentially any cell type can be an antigen-presenting or target cell for CD8+ T-cells, CD4+ T-cells require “professional APC”, such as dendritic cells and B-cells for activation. Therefore, when studying CD8+ T-cell recall responses, the likelihood of detecting every antigen-specific CD8+ T-cell under standard ELISPOT assay conditions is non-restrictive. Subsequently, our data clearly show that within replicate wells, CD8+ T-cell recall responses follow a normal distribution, and in the case of week responses, increasing cell numbers reduces the relative error within replicates. For the aforementioned reasons, this rule may or may not apply for CD4+ T-cells, and this topic is under ongoing investigation in our laboratory.

## 4. Conclusions

Using statistical analysis of spot counts in large numbers of replicate wells, we have shown that ELISPOT counts follow a normal distribution function. The normality of the ELISPOT data enables high power statistical acceptance criteria (Student’s *t*-test, ANOVA) for positive responses. It also allows for predictions of the number of replicate wells needed to detect weak positive responses with known significance and power. Theoretical predictions based on the normal distribution correlated strongly with the experimental observations studying spot counts from weak CD8+ T-cell IFN-γ responses against the subdominant EBV peptide and IFN-γ-transfected CHO cells. We observed that the well-to-well coefficient of variation decreases with increasing numbers of PBMC plated per well. We also showed experimentally that borderline responses can be reliably detected using fewer replicate wells, by plating higher numbers of PBMC, by the addition of IL-7 or a combination of these.

## References

[1] Kuerten S., Batoulis H., Recks M.S., Karacsony E., Zhang W., Subbramanian R.A., Lehmann P.V. (2012). Resting of cryopreserved PBMC does not generally benefit the performance of antigen-specific T-cell ELISPOT assays. Cells.

[2] Ramachandran H., Laux J., Moldovan I., Caspell R., Lehmann P.V., Subbramanian R.A. (2012). Optimal thawing of cryopreserved peripheral blood mononuclear cells for use in high-throughput human immune monitoring studies. Cells.

[3] Zhang W., Lehmann P.V. (2012). Objective, user-independent ELISPOT data analysis based on scientifically validated principles. Methods Mol. Biol..

[4] Mogg R., Fan F., Li X., Dubey S., Fu T.M., Shiver J., Mehrotra D. (2003). Statistical cross-validation of Merck’s IFN-γ ELISPOT assay positivity criterion. Presented at AIDS Vaccine Conference.

[5] Dubey S., Clair J., Fu T.M., Guan L., Long R., Mogg R., Anderson K., Collins K.B., Gaunt C., Fernandez V.R. (2007). Detection of HIV vaccine-induced cell-mediated immunity in HIV-seronegative clinical trial participants using an optimized and validated Enzyme-Linked ImmunoSpot assay. J. Acquir. Immune Defic. Syndr..

[6] Thakur A., Pedersen L.E., Jungersen G. (2012). Immune markers and correlates of protection for vaccine induced immune responses. Vaccine.

[7] McCutcheon M., Wehner N., Wensky A., Kushner M., Doan S., Hsiao L., Calabresi P., Ha T., Tran T.V., Tate K.M. (1997). A sensitive ELISPOT assay to detect low-frequency human T lymphocytes. J. Immunol. Methods.

[8] Hudgens M.G., Self S.G., Chiu Y.L., Russell N.D., Horton H., McElrath M.J. (2004). Statistical considerations for the design and analysis of the elispot assay in HIV-1 vaccine trials. J. Immunol. Methods.

[9] Efron B., Tibshirani R. (1993). An Introduction to the Bootstrap.

[10] Moodie Z., Huang Y., Gu L., Hural J., Self S.G. (2006). Statistical positivity criteria for the analysis of ELISPOT assay data in HIV-1 vaccine trials. J. Immunol. Methods.

[11] Moodie Z., Price L., Gouttefangeas C., Mander A., Janetzki S., Lower M., Welters M.J., Ottensmeier C., van der Burg S.H., Britten C.M. (2010). Response definition criteria for ELISPOT assays revisited. Cancer Immunol. Immunother..

[12] Moodie Z., Price L., Janetzki S., Britten C.M. (2012). Response determination criteria for elispot: Toward a standard that can be applied across laboratories. Methods Mol. Biol..

[13] Dittrich M., Lehmann P.V. (2012). Statistical analysis of ELISPOT assays. Methods Mol. Biol..

[14] Hsu J.C. (1996). Multiple comparisons: Theory and Methods.

[15] Hochberg Y., Benjamini Y. (1990). More powerful procedures for multiple significance testing. Stat. Med..

[16] Westfall P.H., Young S.S. (1993). Resampling-Based Multiple Testing: Exapmples and Methods for p-Value Adjustment.

[17] Hochberg Y., Westfall P.H. (2000). On some multiplicity problems and multiple comparison procedures in biostatistics. Handbook of Statistics.

[18] Huang X.L., Fan Z., Kalinyak C., Mellors J.W., Rinaldo C.R. (2000). Cd8(+) T-cell gamma interferon production specific for Human Immunodeficiency Virus type 1 (HIV-1) in HIV-1-infected subjects. Clin. Diagn. Lab. Immunol..

[19] Nagorsen D., Keilholz U., Rivoltini L., Schmittel A., Letsch A., Asemissen A.M., Berger G., Buhr H.J., Thiel E., Scheibenbogen C. (2000). Natural T-cell response against MHC class I epitopes of epithelial cell adhesion molecule, Her-2/Neu, and carcinoembryonic antigen in patients with colorectal cancer. Cancer Res..

[20] Kuerten S., Schlingmann T.R., Rajasalu T., Angelov D.N., Lehmann P.V., Tary-Lehmann M. (2008). Lack of disease specificity limits the usefulness of *in vitro* costimulation in HIV- and HCV-infected patients. Clin. Dev. Immunol..

[21] SPSS Statistics http://www-01.ibm.com/software/analytics/spss/products/statistics.

[22] XL Stat http://www.xlstat.com.

[23] Hanson J., Sundararaman S., Caspell R., Karacsony E., Karulin A.Y., Zhang W., Lehmann P.V. (2015). Elispot assays in 384 well format: Testing 30 data points with only 1 million cells. Cells.

[24] Royston J.P. (1982). An extension of Shapiro and Wilk’s W tests for normality to large samples. Appl. Stat..

[25] Razali N.M., Wah Y.B. (2011). Power comparison of shapiro-wilk, kolmogorov-smirnov, lilliefors and anderson-darling tests. J. Stat. Model. Anal..

[26] Inference for Means, Comparing Two Independent Samples. http://www.stat.ubc.ca/~rollin/stats/ssize/n2.html.

[27] PBMC Searchable Database http://epbmc.immunospot.com.

